# The role of the folate pathway in pancreatic cancer risk

**DOI:** 10.1371/journal.pone.0193298

**Published:** 2018-02-23

**Authors:** Shirisha Chittiboyina, Zhongxue Chen, E. Gabriela Chiorean, Lisa M. Kamendulis, Barbara A. Hocevar

**Affiliations:** 1 Department of Environmental Health, School of Public Health, Indiana University, Bloomington, Indiana, United States of America; 2 Department of Epidemiology and Biostatistics, School of Public Health, Indiana University, Bloomington, Indiana, United States of America; 3 University of Washington, Fred Hutchinson Cancer Research Center, Seattle, Washington, United States of America; 4 Indiana University Melvin and Bren Simon Cancer Center, Indianapolis, Indiana, United States of America; University of Nebraska Medical Center, UNITED STATES

## Abstract

**Background:**

Pancreatic cancer is the third leading cause of cancer related deaths in the United States. Several dietary factors have been identified that modify pancreatic cancer risk, including low folate levels. In addition to nutrition and lifestyle determinants, folate status may be influenced by genetic factors such as single nucleotide polymorphisms (SNPs). In the present study, we investigated the association between folate levels, genetic polymorphisms in genes of the folate pathway, and pancreatic cancer.

**Methods:**

Serum and red blood cell (RBC) folate levels were measured in pancreatic cancer and control subjects. Genotypes were determined utilizing Taqman probes and SNP frequencies between cases and controls were assessed using Fisher’s exact test. Logistic regression was used to estimate the odds ratio (OR) and corresponding 95% confidence intervals (CIs) to measure the association between genotypes and pancreatic cancer risk. The association between folate levels and SNP expression was calculated using one-way ANOVA.

**Results:**

Mean RBC folate levels were significantly lower in pancreatic cancer cases compared to unrelated controls (508.4 ± 215.9 ng/mL vs 588.3 ± 229.2 ng/mL, respectively) whereas serum folate levels were similar. Irrespective of cancer status, several SNPs were found to be associated with altered serum folate concentrations, including the D919G SNP in methionine synthase (MTR), the L474F SNP in serine hydroxymethyl transferase 1 (SHMT1) and the V175M SNP in phosphatidyl ethanolamine methyltransferase (PEMT). Further, the V allele of the A222V SNP and the E allele of the E429A SNP in methylene tetrahydrofolate reductase (MTHFR) were associated with low RBC folate levels. Pancreatic cancer risk was found to be significantly lower for the LL allele of the L78R SNP in choline dehydrogenase (CHDH; OR = 0.29; 95% CI 0.12–0.76); however, it was not associated with altered serum or RBC folate levels.

## Introduction

Pancreatic cancer, the third leading cause of cancer deaths in the United States, is an aggressive cancer with median 5 year survival rates of only 8% [1]. Detection late in the disease course, rapid metastasis, and chemo-resistance contribute to the poor prognosis for pancreatic cancer [2]. In the age of personalized medicine, identification of genetic and environmental factors that affect the risk for development of pancreatic cancer may aid in prevention or lead to increased surveillance of susceptible individuals. Environmental factors including tobacco and alcohol use, exposure to selected environmental chemicals, obesity, and diet, have been postulated to play a significant role in the etiology of sporadic pancreatic cancer [3]. Deficiencies in dietary sources of methyl groups, including choline, methionine, vitamin B-12 and folate, have been associated with pancreatic dysfunction in rodents [4,5]. In addition, risk of development of various cancer types in humans, including pancreatic cancer, has been shown to increase with low dietary folate intake [6–9].

**Fig 1:**
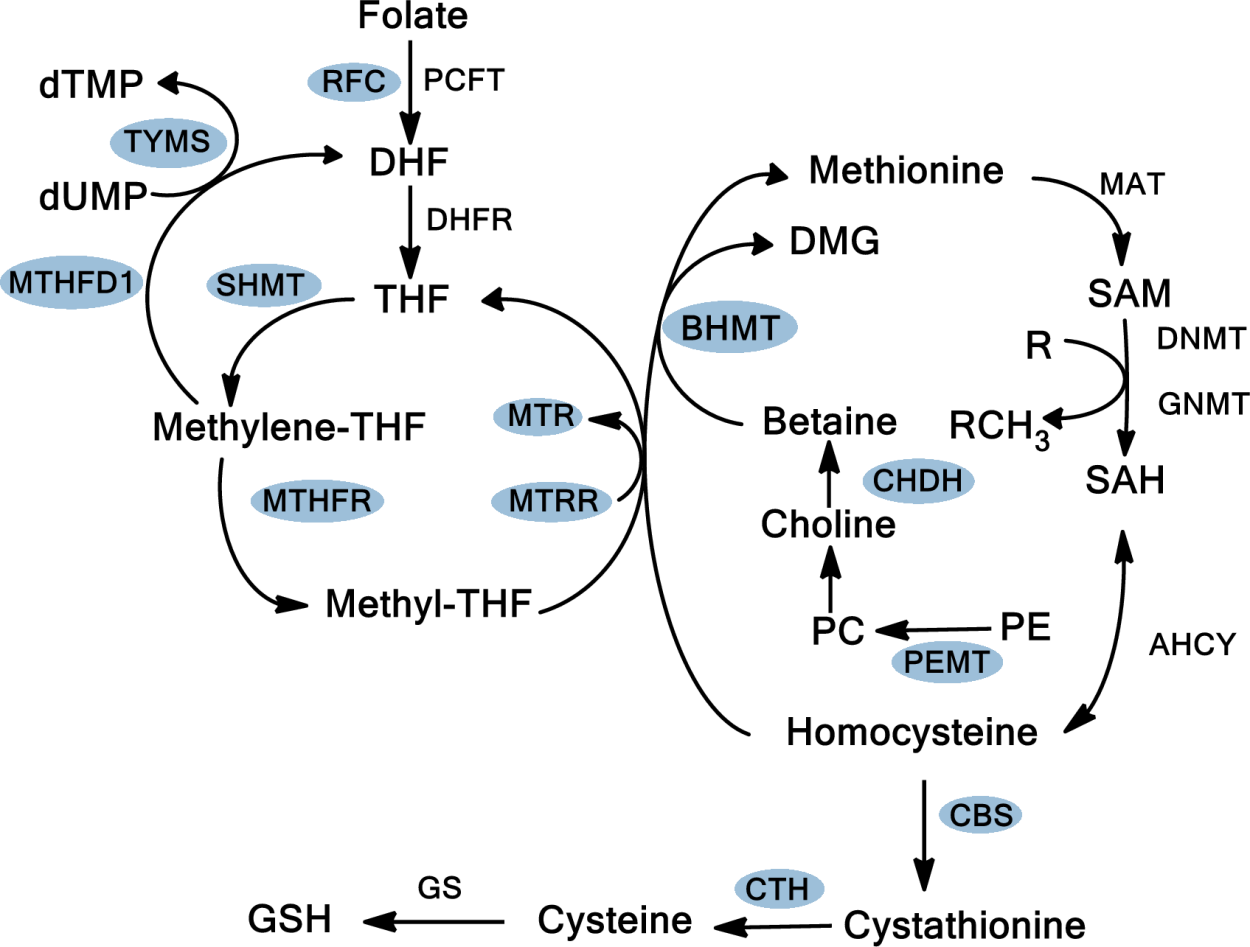
Folate metabolic pathway. Depiction of folate uptake and reduction, transmethylation, and transulfuration pathways. Genes in the shaded circles were analyzed for single nucleotide polymorphisms. RFC, reduced folate carrier; PCFT, proton coupled folate transporter; DHF, dihydrofolate; THF, tetrahydrofolate; DHFR, dihydrofolate reductase; SHMT, serine hydroxymethyltransferase; MTHFR, methylene tetrahydrofolate reductase; MTHFD1, methylene tetrahydrofolate dehydrogenase 1; TYMS, thymidylate synthase; MTR, methionine synthase; MTRR, methionine synthase reductase; BHMT, betaine hydroxymethyl transferase; CHDH, choline dehydrogenase; PC, phosphatidylcholine; PE, phosphatidylethanolamine; PEMT, phosphatidylethanolamine N-methyltransferase; SAM, S-adenosyl methionine; MAT, methionine adenosyltransferase; SAH, S-adenosyl homocysteine; AHCY, S-adeonsyl-L-homocysteine hydrolase; GSH, glutathione; CBS, cystathione ß-synthase; CTH, cystathionase; GS, glutathione synthase.

In humans, folate provides methyl groups for *de novo* deoxynucleotide synthesis and for intracellular methylation reactions. Methylene tetrahydrofolate reductase (MTHFR) plays a central role in folate metabolism by catalyzing the conversion of 5,10-methylenetetrahydrofolate (5,10-methylene THF) to 5-methyltetrahydrofolate (5-methyl THF), which is the main circulating form of folate in the blood and a cosubstrate for the conversion of homocysteine to methionine (Fig 1). Low levels of 5,10-methylene THF can cause an increased dUMP/dTMP ratio, which could result in the incorporation of uracil into DNA in place of thymine, leading to an increased risk for DNA mutations and DNA strand breakage [10]. In addition, low levels of 5-methyl THF can lead to decreased s-adenosylmethionine (SAM) levels, which could result in DNA hypomethylation leading to activation of cellular oncogenes, genomic instability, and DNA damage [11,12]. Low folate levels therefore would be predicted to modify cancer risk by influencing both pathways.

Disruption of the folate metabolic pathway can result from either insufficient dietary intake of folate or the presence of genetic variants, such as single nucleotide polymorphisms (SNPs), in genes of the folate pathway. Several SNPs in genes of the folate pathway have been shown to result in altered serum and red blood cell (RBC) folate levels, including SNPs in the reduced folate carrier (RFC) [13], dihydrofolate reductase (DHFR) [14] and MTHFR genes [15]. An increased risk for development of pancreatic cancer has been associated with expression of the VV allele of the A222V SNP in MTHFR [16–19]. In addition, expression of the YY genotype in the methionine synthase reductase (MTRR) H595Y SNP and the 3Rc/3Rc genotype in thymidylate synthase (TYMS) also increased the risk for development of pancreatic cancer [18,20].

We have previously designed a case-control study to investigate the contribution of environment and genetics to the risk for development of pancreatic cancer, where we recruited pancreatic cancer subjects, an unrelated control who resided with the subject, as well as a genetic relative control who did not reside with the subject [21]. Initial results from this study demonstrated that direct DNA damage was significantly higher in pancreatic cancer patients as compared to healthy related and unrelated controls [21]. As the folate pathway plays a supportive role in DNA synthesis and repair pathways, the overall goal of this study was to investigate whether altered folate levels are observed in these patients and whether expression of SNPs in genes of the folate metabolic pathway are associated with pancreatic cancer risk.

## Materials and methods

### Ethics statement

This study was approved by the Indiana University Institutional Review Board. A signed written informed consent form was obtained from all participants at the time of recruitment.

### Study population

A total of 265 subjects were recruited, of which 159 were pancreatic cancer cases (Stage I-IV), 55 were unrelated controls (spouses or partners) who shared the same environment, and 51 were related controls (children or siblings) who were expected to share a similar genetic makeup. Stratification of the controls was chosen such that the contribution of both environmental and genetic factors and their interaction in pancreatic cancer risk could be investigated. Genetically related controls were included provided that they did not reside with the case whereas unrelated controls were included only if they cohabited with the case. All subjects were Caucasian, >18yrs of age, and able to read, understand and sign a written informed consent. Information on demographics was collected through a questionnaire. Cases with any other malignancies besides pancreatic cancer, who had previously received chemotherapy, or any other treatment for cancer were excluded. Blood samples for isolation of genomic DNA and serum and RBC folate levels were collected from all participants at the time of recruitment.

### DNA extraction and SNP genotyping

Whole blood was collected and stored in vacutainer vials (BD Diagnostics, Franklin Lakes, NJ) at -80°C until further analysis. Genomic DNA was isolated from peripheral blood mononuclear cells (PBMCs) using the QIAamp DNA blood midi kit (Qiagen, Valencia, CA). Allelic discrimination was performed using fluorogenic SNP Taqman probes (Applied Biosystems, Carlsbad, CA) for selected SNPs in the folate pathway (S1 Table) and analyzed on an Illumina Eco Real-Time PCR machine (Illumina, San Diego, CA). Genotypes were assigned using the analysis software provided by Illumina.

### Microbiological assay for folate determination

Folate levels were measured in serum or plasma and RBCs using the *Lactobacillus casei* microbiological assay [22,23]. *Lactobacillus casei*, a folate-dependent bacterium whose growth is an indirect indicator of folate levels in a sample, was purchased from ATCC (ATCC 7946; ATCC, Manassas, VA), and cultured in *L*.*casei* broth (BD Biosciences), which contains minimal levels of folate. Bacterial stocks were prepared and cryopreserved in glycerol at -80°C. To quantify folate levels in RBCs, whole blood samples were lysed with 1% ascorbic acid and further diluted in 0.5% sodium ascorbate. For the microbiological assay, cryopreserved bacteria were suspended in 0.9% NaCl and added to folate standards and the serum/plasma or whole blood samples. After incubation in a rotating shaker at 37°C for 48 hrs, absorbance of the cultures was determined at 546 nm, which was used as a measure of bacterial growth. Serum/plasma and whole blood folate concentrations were determined using polynomial regression generated from a standard curve. To calculate RBC folate levels, hemoglobin was measured using the whole blood samples as per the manufacturer’s protocol (Hemoglobin Assay kit, Sigma). The hemoglobin concentrations were then used to calculate hematocrit (Hct) levels from which RBC folate levels were derived according to the formula:

$$RBC_{folate}=Whole\;blood_{folate}\;*\;dilution\;factor–\left[serum_{folate}\;\left(1-Hct/100\right)\right]/\left(Hct/100\right)].$$

### Statistical analysis

Fisher’s exact test was used to compare SNP frequencies between patients and unrelated healthy controls. Logistic regression was used to estimate odds ratios (OR) and corresponding 95% confidence intervals to measure the association between genotypes and risk for pancreatic cancer. Hardy-Weinberg Equilibrium (HWE) was tested using χ ^2^ analysis. The gene dose overall, dominance and recessive were calculated using χ ^2^ analysis. To measure the correlation between folate levels and genotype, the independent samples t-test was used. One-way ANOVA was used to calculate overall p-value for the association of genotype with serum and RBC folate levels. Serum and RBC folate levels were compared between patients and controls (unrelated and related) using the Student’s t-test. Serum tertile distribution among patients and controls was analyzed using the Student’s t-test. The serum tertile distribution was based on the CDC definition of deficient (≤2.6 ng/mL), normal (2.6–14.2 ng/mL) and above normal (≥14.2 ng/mL) serum folate ranges (Centers for Disease Control and Prevention www.cdc.gov). For RBC folate stratification, the deficient and normal range distribution was combined because of insufficient subjects in the deficient range according to the CDC definition of normal (102.6–410.9 ng/mL), deficient (≤ 102.6 ng/mL) and above normal (≥ 410.9 ng/mL) RBC folate concentrations (Centers for Disease Control and Prevention www.cdc.gov). Age at the time of diagnosis for the cancer cases, age at the time of sample collection, and date for control groups was included as a continuous variable. Gender for the study population was included as a dichotomous variable. The resulting ORs and corresponding CIs were reported both unadjusted and adjusted for age and gender due to differences in these variables among the patients and the controls. P-values of <0.05 were considered statistically significant. All statistical analyses were performed using SPSS 16.0.

## Results

### Demographic characteristics of the study population

Age and gender of the study population are shown in Table 1. Pancreatic cancer cases were significantly older than healthy related controls (p = 0.002); however, the unrelated controls exhibited a similar age range as the cases. In this study, pancreatic cancer cases were more likely to be males.

**Table 1:** Demographics of the subject population.

	Pancreatic cancer cases(n = 159)	Healthy unrelated controls (n = 55)	Healthy related controls (n = 51)	p-value
Age^a^				
Mean ± SD	64.2 ± 11.5	60.1 ± 11.6	46.6 ± 11.8	0.002^b^
Range	23–92	33–87	19–80	
Gender^a^				
Male	82 (55%)	13 (26%)	16 (32%)	0.0003^c^
Female	68 (45%)	37 (74%)	34 (68%)	

^a^Missing information for 9 cases, 5 healthy unrelated controls and 3 healthy related controls

^b^Comparison between cases and healthy unrelated controls

^c^Comparison of gender in cases only

### Association of serum and RBC folate levels to pancreatic cancer

Serum folate levels were not significantly different between pancreatic cancer cases (9.5 ± 4.9 ng/mL) and unrelated (9.3 ± 4.0 ng/mL) or related controls (9.6 ± 4.5 ng/mL) (Table 2). However, while not achieving statistical significance (p = 0.05), 12% of the pancreatic cancer cases exhibited deficient serum folate levels (below 2.6 ng/mL) compared with only 7% of the healthy unrelated control group (S2 Table). Pancreatic cancer cases exhibited significantly lower RBC folate levels (508.4 ± 215.9 ng/mL) compared to unrelated controls (588.3 ± 229.2 ng/mL); however, RBC folate levels in the related controls were not significantly different from that in the cases (Table 2). When RBC folate levels were stratified according to CDC criteria, 33% of the cases demonstrated deficient to normal RBC folate levels (<102.6–410.9 ng/mL) whereas 67% were observed to have higher than normal (>410.9 ng/mL) RBC folate levels. However, these values were not statistically different between cases and controls (S3 Table).

**Table 2:** Serum and RBC folate levels in pancreatic cancer cases and controls.

	n*	Serum folate^a^ (ng/mL)	p-value	RBC folate^a^ (ng/mL)	p-value
Pancreatic cancer cases	143	9.5 ± 4.9	—	508.4 ± 215.9	—
Unrelated controls	45	9.3 ± 4.0	0.40	588.3 ± 229.2	0.01^#^
Related control	48	9.6 ± 4.5	0.43	531.8 ± 239.7	0.20

*Missing serum samples for 16 cases; 10 unrelated controls and 3 related controls

^a^Results are expressed as mean ± SD

^#^Significantly different, p<0.05

### Association of SNPs in the folate pathway and pancreatic cancer

Genotypes for 15 SNPs in 12 genes of the folate metabolic pathway (Fig 1 and S1 Table) were determined in the pancreatic cancer cases and control groups (Table 3). A significant difference was observed in the genotype distribution among patients and healthy unrelated controls for the choline dehydrogenase CHDH L78R SNP (p = 0.03), as well as in the dominant gene dose model (p = 0.02) (Table 3). No other significant differences were observed in genotype frequencies for the remaining SNPs in the folate pathway. Hardy-Weinberg equilibrium (HWE) was next tested using χ^2^ analysis for these SNPs and polymorphisms that did not follow HWE were not analyzed further. Odds ratios were determined for the remaining 8 SNPs (Table 4). Subjects that carried the LL genotype (nucleotide TT) of the CHDH L78R SNP were found to be at decreased risk for pancreatic cancer both prior to (OR = 0.29; 95% CI 0.12–0.76) and after adjusting for age and sex (OR = 0.29; 95% CI 0.10–0.84) (Table 4). No other significant associations with pancreatic cancer risk for the remaining SNPs in the folate pathway were observed.

**Table 3:** Genotype frequencies of SNPs in genes in the folate pathway.

				Amino	Cases	Unrelated	p-values		
rs^#^	Gene	SNP	Genotype	Acid	n (%)	Controls n (%)	Overall	Dominance	Recessive
3578659000	RFC	A558V	TT	AA	65 (100)^#^	31 (100)^#^	1.00	1.00	1.00
			CT	AV	0 (0)	0 (0)			
			CC	VV	0 (0)	0 (0)			
1979277	SHMT	L474F	CC	LL	66 (42)	26 (47)	0.77	0.43	0.28
			CT	LF	78 (49)	25 (45)			
			TT	FF	15 (9)	4 (8)			
2236225	MTHFD1	Q653R	GG	RR	45 (28)	15 (27)	0.95	0.52	0.23
			AG	QR	82 (52)	28 (51)			
			AA	QQ	32 (20)	12 (22)			
59755869	TYMS	E100Q	CC	EE	0 (0)	0 (0)	1.00	1.00	1.00
			CG	EQ	0 (0)	0 (0)			
			GG	QQ	71 (100)^#^	31 (100^#^			
1801133	MTHFR	A222V	CC	AA	61 (38)	24 (44)	0.30	0.16	0.30
			CT	AV	73 (46)	26 (47)			
			TT	VV	25 (16)	5 (9)			
1801131	MTHFR	E429A	AA	EE	87 (54)	24 (44)	0.25	0.20	0.08
			AC	EA	60 (38)	24 (44)			
			CC	AA	12 (8)	7 (12)			
10380	MTRR	H595Y	CC	HH	123 (77)	46(84)	0.32	0.55	0.22
			CT	HY	34 (21)	9 (16)			
			TT	YY	2 (1)	0 (0)			
1805087	MTR	D919G	AA	DD	101 (64)	34 (62)	0.88	0.55	0.47
			AG	DG	45 (28)	17 (31)			
			GG	GG	13 (8)	4 (7)			
12749581	MTR	R52Q	GG	RR	59 (33)	18 (33)	0.63	0.34	1.00
			AG	RQ	100 (67)	37 (67)			
			AA	QQ	0 (0)	0 (0)			
3733890	BHMT	Q239R	GG	RR	81 (51)	27 (49)	0.87	0.47	0.35
			AG	RQ	67 (42)	23 (42)			
			AA	QQ	11 (7)	5 (9)			
7946	PEMT	V175MMMM	TT	MM	88 (55)	35 (64)	0.27	0.30	0.23
			CT	MV	55 (35)	17 (31)			
			CC	VV	16 (10)	3 (5)			
9001	CHDH	E40A	AA	EE	124 (78)	46 (83)	0.47	0.70	0.24
			AC	EA	34 (21)	9 (17)			
			CC	AA	1 (1)	0 (0)			
12676	CHDH	L78R	GG	RR	84 (53)	21 (38)	0.03*	0.02*	0.06
			GT	LR	62 (39)	23 (42)			
			TT	LL	13 (8)	11 (20)			
1021737	CTH	S430I	GG	SS	85 (53)	27 (49)	0.59	0.33	0.34
			GT	SI	54 (35)	23 (42)			
			TT	II	20 (12)	5 (9)			
234706	CBS	C699T	CC		0 (0)	0 (0)	1.00	1.00	1.00
			CT		159 (100)	55 (100)			
			TT		0 (0)	0 (0)			

^#^ Not all subjects were analyzed as genotypes were all the same

*Significantly different, p<0.05

**Table 4:** Association of folate metabolic gene SNPs and pancreatic cancer.

	Amino Acid	Genotype	Cases n (%)	Unrelated Controls n (%)	UnadjustedOR (95% CI)	p-value	AdjustedOR (95% CI)	p-value
SHMT L474F	LL	CC	66 (42)	26 (47)	1.00	-		
	LF	CT	78 (49)	25 (45)	0.68 (0.20–2.23)	0.52	1.23 (0.61–2.48)	0.57
	FF	TT	15 (9)	4(8)	0.83 (0.25–2.74)	0.76	1.46 (0.41–5.21)	0.56
MTHFD1 Q653R	RR	GG	45 (28)	15 (27)	1.00			
	QR	AG	82 (52)	28 (51)	0.59 (0.25–1.35)	0.21	1.63 (0.28–1.77)	0.29
	QQ	AA	32 (20)	12 (22)	0.89 (0.37–2.15)	0.79	1.07 (0.59–2.06)	0.78
MTHFR A222V	AA	CC	61(38)	24 (44)	1.00			
	AV	CT	73 (46)	26 (47)	0.61 (0.22–1.67)	0.33	1.11 (0.54–2.31)	0.78
	VV	TT	25 (16)	5 (9)	0.70 (0.25–1.90)	0.49	1.32 (0.45–3.82)	0.62
MTHFR E429A	EE	AA	87 (54)	24 (44)	1.00	-		
	EA	AC	60 (38)	24 (44)	1.93 (0.69–5.37)	0.21	0.63 (0.31–1.31)	0.22
	AA	CC	12 (8)	7 (12)	1.35 (0.47–3.78)	0.57	0.58 (0.19–1.76)	0.34
MTR D919G	DD	AA	101 (64)	34 (62)	1.00			
	DG	AG	45 (28)	17 (31)	0.91 (0.28–2.99)	0.88	0.66(0.32–1.39)	0.28
	GG	GG	13 (8)	4 (7)	0.81 (0.23–2.85)	0.74	1.30 (0.32–5.29)	0.72
PEMT V175M	MM	TT	88 (55)	35 (64)	1.00	-		
	VM	CT	55 (35)	17 (31)	1.29 (0.66–2.52)	0.46	1.62 (0.68–8.46)	0.61
	VV	CC	16 (10)	3 (5)	2.12 (0.58–7.74)	0.24	2.64 (0.83–10.61)	0.39
BHMT Q239R	RR	GG	81 (51)	27 (49)	1.00			
	QR	AG	67 (42)	23 (42)	0.97 (0.51–1.84)	0.93	0.78 (0.38–1.57)	0.48
	QQ	AA	11 (7)	5 (9)	0.73 (0.23–2.30)	0.59	0.77 (0.21–2.79)	0.69
CHDH L78R	RR	GG	84 (53)	21 (38)	1.00			
	RL	GT	62 (39)	23 (42)	0.67 (0.34–1.33)	0.25	0.69 (0.33–1.64)	0.33
	LL	TT	13 (8)	11 (20)	0.29 (0.12–0.76)	0.01*	0.29 (0.10–0.84)	0.02*

*Significantly different, p<0.05

### Association of SNPs in the folate pathway with altered serum and RBC folate levels

We observed significantly different serum folate levels associated with individuals expressing the serine hydroxymethyltransferase (SHMT1) L474F, 5-methltetrahydrofolate-homocysteine methyltransferase (MTR) D919G and the phosphatidylethanolamine N-methyltransferase (PEMT) V175M polymorphisms (Table 5). Individuals with the LF allele of the SHMT1 L474F SNP showed significantly higher serum folate levels (10.4 ± 0.4 ng/mL) compared to those of LL allele (9.1 ± 0.4 ng/mL); however a gene dose-response trend was not observed. Individuals with the DG genotype of the MTR D919G polymorphism had significantly lower serum folate concentrations (8.2 ± 0.5 ng/mL) compared to those expressing the reference genotype DD (10.3 ± 0.3 ng/mL). For the PEMT V175M polymorphism, the subjects with the VV genotype exhibited significantly lower serum folate levels (7.1 ± 1.2 ng/mL) compared to the reference genotype MM (9.6 ± 0.4 ng/mL).

**Table 5:** Association of folate levels with genotype.

SNP	Allele	n	Serum folate (ng/mL)	p-value	RBC folate (ng/mL)	p-value	
SHMT1 L474F	LL	111	9.1 ± 0.4		533.0 ± 20.8		
	LF	101	10.4 ± 0.4	0.03*	515.7 ± 22.9		0.57
	FF	25	7.8 ± 1.4	0.09	559.4 ± 45.7		0.58
MTHFD1 Q653R	RR	51	9.0 ± 0.6		510.4 ± 29.9		
	QR	124	9.7 ± 0.4	0.69	547.0 ± 20.5		0.37
	QQ	62	9.4 ± 0.6	0.72	505.5 ± 28.1		0.21
MTHFR E429A	EE	120	10.0 ± 0.4		496.6 ± 21.5		
	EA	95	8.9 ± 0.5	0.09	560.0 ± 21.5		0.04*
	AA	22	9.1 ± 0.9	0.39	563.8 ± 44.6		0.05
MTHFR A222V	AA	96	8.9 ± 0.5		603.5 ± 21.4		
	AV	108	9.7 ± 0.4	0.09	509.6 ± 21.3		0.002*
	VV	33	10.6 ± 0.7	0.06	370.6 ± 31.7		<0.001*
MTRR H595Y	HH	183	9.4 ± 0.3		531.2 ± 16.9		
	HY	52	9.7 ± 0.6	0.68	526.2 ± 28.5		0.88
	YY	2	11.1 ± 4.0	0.59	322.5 ± 151.5		0.24
MTR D919G	DD	143	10.3 ± 0.3		524.8 ± 18.7		
	DG	72	8.2 ± 0.5	0.002*	520.2 ± 26.9		0.88
	GG	22	8.6 ± 0.9	0.122	578.7 ± 46.6		0.29
BHMT Q239R	RR	127	9.2 ± 0.4		526.5 ± 19.3		
	QR	96	10.1 ± 0.5	0.15	531.6 ± 23.6		0.89
	QQ	14	8.4 ± 1.4	0.54	522.9 ± 67.7		0.95
PEMT V212M	MM	132	9.6 ± 0.4		525.5 ± 18.4		
	VM	84	9.9 ± 0.5	0.70	538.5 ± 26.7		0.58
	VV	21	7.1 ± 1.2	0.03*	506.5 ± 49.8		0.70
CHDH E40A	EE	184	9.2 ± 0.3		541.7 ± 16.3		
	EA	51	10.6 ± 0.6	0.06	526.2 ± 28.5		0.06
	AA	2	8.9 ± 0.4	0.94	676.5 ± 198.8		0.39
CHDH L78R	RR	117	9.41 ± 0.44		510.7 ± 19.8		
	LR	93	9.67 ± 0.46	0.70	548.0 ± 23.9		0.84
	LL	27	9.29 ± 0.92	0.90	538.1 ± 48.3		0.56
CTH S430I	SS	135	9.3 ± 0.4		521.8 ± 19.2		
	SI	81	10.1 ± 0.5	0.25	526.3 ± 25.7		0.88
	II	21	8.5 ± 1.2	0.47	578.6 ± 45.2		0.27

*Significantly different, p<0.05

Significant differences in RBC folate levels were observed in the subjects expressing A222V and E429A polymorphisms of the MTHFR gene. Subjects with the AV and VV genotypes of the MTHFR A222V SNP showed significantly lower RBC folate levels (509.6 ± 21.3 ng/mL and 370.6 ± 31.7 ng/mL respectively) compared to individuals expressing the wild type allele AA (603.5 ± 21.4 ng/mL). The EE allele of the MTHFR E429A polymorphism was also found to be significantly associated with lower RBC folate levels (496.6 ± 21.5 ng/mL) compared to the heterozygous EA and AA variant allele (560.0 ± 21.5 ng/mL and 563.8 ± 44.6 ng/mL, respectively).

## Discussion

Pancreatic cancer is a devastating disease, with a relative 5 year survival rate of 8%. One of the reasons for the dismal prognosis is that more than half of the cases are diagnosed at late stage, where the 5-year survival rate is only 3% [1]. As such, identification of risk susceptibility profiles for pancreatic cancer could greatly impact early diagnosis and prevention strategies. In a pilot study, we have previously shown that direct DNA damage was increased in pancreatic cancer patients as compared to control subjects who resided with the patient (i.e. shared the same environment) and also as compared to controls that are genetically related, but did not share the same environment [21]. As defects in the folate metabolic pathway have been linked to DNA damage, in this study we sought to determine (1) whether serum and RBC folate levels differ between pancreatic cancer patients and controls, (2) whether SNPs in key genes of the folate pathway associate with pancreatic cancer risk and (3) whether expression of specific SNPs correlates with serum or RBC folate levels.

The relationship between dietary folate intake, measured serum and RBC folate levels, and pancreatic cancer risk is not clearly defined. Dietary folate intake has been shown to be inversely proportional to pancreatic cancer risk in several epidemiological studies in which higher dietary folate intake was associated with lower risk for pancreatic cancer [24–26]. However, in the Nurses’ Health Study and the Health Professionals Follow-up Study, no relationship between folate intake and pancreatic cancer risk was observed [27]. Low serum folate levels were associated with increased risk of pancreatic cancer in male Finnish smokers in the Alpha Tocopherol Beta Carotene Cancer prevention (ATBC) study [6]. Interestingly, the European Prospective Investigation Into Cancer study has shown a U-shaped relationship between plasma folate and pancreatic cancer risk; that is, individuals with high plasma folate (>20 nM) and those with deficient or near deficient plasma folate levels (5–10 nM) had a similar increased risk when compared to those with adequate plasma folate levels (10–15 nM) [7]. In our study, we did not find significant differences in the mean serum folate levels of the pancreatic cancer cases and the unrelated controls or related controls (9.5 ± 4.9ng/mL, 9.3 ± 4.0 ng/mL, and 9.6 ± 4.5 ng/mL, respectively) (Table 2). We did observe however, that 12% of cases fell in the deficient range (<2.6 ng/mL) for serum folate, compared to only 6% in unrelated controls and 7% in related controls (S2 Table). Since 1998, the FDA has required folate fortification of cereals and grains in the US, and folate levels in the general population have risen significantly [28], which may explain why differences in serum folate levels were not observed in our study. In addition, the ATBC study consisted exclusively of male smokers and smoking has been associated with low circulating folate levels [29].

Although RBC folate levels have been studied with respect to obesity and neural tube defects, our study is the first, to our knowledge, to determine an association of RBC folate levels with pancreatic cancer risk. We observed that RBC folate levels in pancreatic cancer cases (508.4 ± 215.9 ng/mL) were significantly lower than that of unrelated healthy controls (588.3 ± 229.2 ng/mL), but not that of related controls (531.8 ± 239.7 ng/mL), suggesting that RBC folate levels are, in part, dependent on genetics. Compared to serum folate levels, which are indicative of recent folate intake, RBC folate concentrations represent tissue folate levels, and hence are more representative of an individual’s overall folate status. While the RBC folate levels were lower in pancreatic cancer subjects in this study, the observed levels are considered to be within the CDC’s definition of folate-sufficient status. It is possible that the lower RBC folate in the cases could have been due to underlying pathologies caused by cancer, such as poor folate absorption or increased demand for DNA synthesis. On the other hand, animal studies have shown that folic acid supplementation decreased MTHFR enzymatic activity resulting in reduced methylation potential, suggesting that high tissue folate levels mimic MTHFR deficiency [30]. As 67% of the pancreatic cancer cases were observed to have higher than normal RBC folate levels according to CDC guidelines, further assessment of folate metabolism in pancreatic cancer, (e.g. determination of methyl-THF, SAM and SAH levels) appears warranted.

While the MTHFR A222V, MTRR H595Y, and TYMS 3Rc SNPs have previously been shown to be associated with pancreatic cancer [18,20], the studies did not include measurements of serum or RBC folate levels. In our study, we evaluated gene polymorphisms involved in the uptake, transmethylation and transulfuration pathways of folate metabolism (Fig 1) for their association with pancreatic cancer risk as well as association of folate levels with these gene polymorphisms. The MTHFR A222V polymorphism results from a nucleotide change at position 677 from C to T. Individuals heterozygous (CT) or homozygous (TT) for the C677T polymorphism exhibit lower enzymatic activity, 65% and 30% respectively, compared to CC homozygous individuals [9]. Decreased activity of MTHFR exhibited by the TT genotype is thus predicted to result in DNA hypomethylation and indeed, expression of the VV allele has been associated with decreased DNA methylation compared to the AA allele [31]. DNA hypomethylation, which may lead to aberrant expression of proto-oncogenes or overexpression of proteins involved in cancer progression, has been shown previously in pancreatic cancer [32]. Li et al. reported a 2-fold increased risk for pancreatic cancer for individuals with the MTHFR 677 TT versus the CC/CT polymorphisms while Wang et al. reported increased odds ratios of 2.6 and 5.1 for the CT and TT genotypes, respectively, versus the wild-type CC polymorphism [16,18]. While we did not observe an association of the MTHFR A222V or MTRR H595Y polymorphism with pancreatic cancer, we found that expression of the homozygous variant allele results in lower RBC folate levels (Table 5). Our results are consistent with other studies that observed lower RBC folate levels in individuals with the AV and VV alleles in the MTHFR A222V SNP [31,33,34]. In contrast, individuals with the EA and AA variants of the MTHFR E429A SNP had higher mean RBC folate concentrations as compared to the referent EE genotype. This is consistent with previous observations, in that the ancestral and more common alleles of the MTHFR A222V and MTHFR E429A polymorphisms exhibited opposite effects on RBC folate levels [35]. While we did not find statistically significant associations of pancreatic cancer with either of the MTHFR SNPs, the percentage of pancreatic cancer patients with the VV genotype in the A222V SNP, with the lowest RBC folate levels, is almost double that of unrelated control subjects (16% vs 9%, respectively; Table 3), and the percentage of pancreatic cancer patients with the EE genotype of the E429A SNP, with lower RBC folate levels, is also greater than that of unrelated control subjects (54% vs 44%, respectively; Table 3). A study by Keku et al. found that the AA genotype of the E429A SNP, that is associated with high RBC folate levels, was only protective when dietary folate was low, but not when folate intake was high, suggesting that determination of the genotype of an individual alone may not adequately assess risk [36]. Rather, other factors, such as dietary intake of folate, need to be considered along with genotype. As dietary folate intake data was not collected in our study, we cannot perform these same correlations in this study group. We have observed that the SHMT1 L474F, MTR D919G, and PEMT V175M polymorphisms display significant differences in serum folate levels among the alleles (Table 5). The variant alleles of the SHMT 1 L474F and MTR D919G polymorphisms have been implicated in increased risk of certain cancers [37–39]. We found that individuals expressing the LF allele of the SHMT1 L474F SNP exhibited significantly higher serum folate levels compared to those carrying the referent LL allele; however, serum folate levels in FF homozygous individuals did not significantly differ from those with the LL allele. These results are consistent with previous findings that individuals with the LL genotype exhibited lower plasma folate levels than those of the LF genotype [40]. SHMT1 catalyzes the reaction for generation of methylene-THF, which can either be utilized in thymidylate or methionine biosynthesis. While the amino acid change of leucine to phenylalanine does not appear to alter enzymatic activity, it impairs nuclear transport of SHMT1 resulting in cytosolic accumulation of the altered SHMT1 where it is not able to facilitate thymidylate synthesis [41–43]. As such, one might predict that expression of this SNP would confer a protective effect against development of pancreatic cancer; however, expression of a cytoplasmic isozyme of SHMT2, SHMT2α, appears to functionally compensate for lack of wild-type SHMT1 protein [41].

The enzyme MTR catalyzes the remethylation of homocysteine to methionine (Fig 1). The functional impact of the MTR D919G polymorphism is not known, however it has been postulated to alter enzyme activity [38]. The presence of the G allele in the MTR D919G SNP has been associated with lower plasma homocysteine levels and higher folate concentrations as compared to DD genotype in the Physicians Health Study (PHS) [44]. In contrast, we found that the G allele was associated with significantly lower serum folate levels compared to that of the DD genotype (Table 5). This could be due to the difference in the mean serum folate ranges observed in these studies, as the PHS study was conducted before folate fortification in the US. PEMT is a key enzyme in the choline metabolic pathway converting phosphatidylethanolamine (PE) to phophatidylcholine (PC) which is further converted to choline. Choline is the substrate for betaine synthesis which in turn provides the methyl group for methionine synthesis (Fig 1). PEMT therefore, plays a key role in the methylation cycle of the folate metabolic pathway. The amino acid substitution of methionine for valine at position 175 in PEMT (V175M) results in production of an enzyme with reduced activity [45]. In our study, we found that individuals with the VV genotype of the V175M PEMT SNP exhibited significantly lower serum folate levels than those with the MM genotype. In concordance with these findings, women with the rarer VV genotype were more likely to have a child with a neural tube defect [46].

Our study is the first to report a significant association between the L78R CHDH SNP and the risk of pancreatic cancer, where we found that the LL genotype of the polymorphism L78R CHDH was significantly protective (OR = 0.29; CI 0.12–0.76) against pancreatic cancer when compared with the referent RR allele. The CHDH enzyme catalyzes the synthesis of betaine from choline. In addition to 5,10 methylene THF, betaine is an alternative substrate for methionine synthesis, which would aid in maintaining sufficient pools of SAM for DNA methylation reactions. While detailed investigation of the effect of this SNP on CHDH enzyme activity has not been conducted, several studies suggest that the L78R substitution results in a functional change of the protein. Expression of the minor T allele, which codes for expression of leucine, has been associated with development of clinical symptoms of choline deficiency [47]. In addition, infertility linked to altered sperm function was found in men with the GT or TT genotype, which was attributed to decreased ATP production by dysmorphic mitochondria [48]. Further, CHDH protein levels in sperm and hepatocytes in individuals with either the GT and TT genotype was decreased in comparison to that of the GG individuals [48]. As folate levels were not different among genotypes in the L78R SNP (Table 5), the mechanism by which the TT allele confers protection against pancreatic cancer is likely not due to modulation of folate metabolism. CHDH has recently been found to stimulate mitophagy, a form of autophagy, following mitochondrial damage [49]. As autophagy has been shown to promote pancreatic cancer [50], we postulate that the inability of the L78 form of CHDH to stimulate autophagy may lead to protection against pancreatic cancer development. Further studies are required to validate this hypothesis.

One limitation of our study is the small size of the study population, which may have affected the statistical power of our study and may explain inconsistencies seen with previous larger epidemiological studies. Another limitation is the lack of information on lifestyle factors such as smoking, alcohol consumption, and dietary folate intake which can influence folate status. In conclusion, in this study, we have explored several SNPs in genes in the folate pathway for their association with both folate status and pancreatic cancer, and have identified that the LL variant of the L78R SNP in CHDH is associated with a decreased risk for pancreatic cancer. Further studies are needed to verify and extend these findings in larger pancreatic cancer cohorts.

## Supporting information

S1 Table SNPs in the folate pathway examined in this study. (DOCX)

S2 Table Serum folate tertile distribution among patients and controls. (DOCX)

S3 Table Red blood cell folate tertile distribution among patients and controls. (DOCX)
