# Supplementary material for: The role of the folate pathway in pancreatic cancer risk
Source: PLoS One. 2018 Feb 23;13(2):e0193298. doi: 10.1371/journal.pone.0193298 (PMC5825090; doi:10.1371/journal.pone.0193298)
Supplement: S1 Table — (DOCX) [file pone.0193298.s001.docx]

S1 Table: SNPs in the folate pathway examined in this study

| rs # | Gene | SNP | ABI Catalog # |
| --- | --- | --- | --- |
| 35786590 | RFC | A558V | C__25762142_20 |
| 1979277 | SHMT | L474F | C__3063127_10 |
| 2236225 | MTHFD1 | Q653R | C__1376137_10 |
| 59755869 | TS | E100Q | C__786091_10 |
| 1801131 | MTHFR | E429A | C__850486_20 |
| 1801133 | MTHFR | A222V | C__1202883_20 |
| 10380 | MTRR | H595Y | C__7580070_1 |
| 1805087 | MTR | D919G | C__12005959_10 |
| 12749581 | MTR | R52Q | C__31400642_10 |
| 3733890 | BHMT | Q239R | C__11646606_20 |
| 7946 | PEMT | V175M | C__9245965_10 |
| 9001 | CHDH | E40A | C__7553897_10 |
| 12676 | CHDH | L78R | C__11741767_10 |
| 1021737 | CTH | S430I | C__8369524_10 |
| 234706 | CBS | C699T | C__1605443_10 |
