# Supplementary material for: The role of the folate pathway in pancreatic cancer risk
Source: PLoS One. 2018 Feb 23;13(2):e0193298. doi: 10.1371/journal.pone.0193298 (PMC5825090; doi:10.1371/journal.pone.0193298)
Supplement: S2 Table — (DOCX) [file pone.0193298.s002.docx]

S2 Table: Serum folate tertile distribution among patients and controls

| Folate conc.  (ng/mL) | Cases  n (%) | Unrelated controls  n (%) | Related control  n (%) | p |
| --- | --- | --- | --- | --- |
| ≤2.6ng/ml(deficient) | 17 (12) | 3 (7) | 3 (6) |  |
| 2.6-12.2(normal) | 65 (45) | 28 (62 ) | 26 (54) |  |
| ≥12.2(above normal) | 61 (43) | 12 (31) | 19 (40) |  |
| p-value |  | 0.05^a^ | 0.23^b^ | 0.12^c^ |

^a^ p-value for serum folate tertile distribution between cases and unrelated controls; ^b^ p-value for serum folate tertile distribution between cases and related controls; ^c^ p-value for serum folate tertile distribution among case, unrelated controls and related controls.
