# Supplementary material for: The role of the folate pathway in pancreatic cancer risk
Source: PLoS One. 2018 Feb 23;13(2):e0193298. doi: 10.1371/journal.pone.0193298 (PMC5825090; doi:10.1371/journal.pone.0193298)
Supplement: S3 Table — (DOCX) [file pone.0193298.s003.docx]

S3 Table: Red blood cell folate tertile distribution among patients and controls

| Folate conc  (ng/mL) | Cases n (%) | Unrelated controls  n (%) | Related controls  n (%) | p |
| --- | --- | --- | --- | --- |
| <102.6 - 410.9 | 47 (33) | 15 (27) | 16 (33) |  |
| >410.9 | 96 (67) | 40 (73) | 32 (67) |  |
| p-value |  | 0.44^a^ | 1.00^b^ | 0.57^c^ |

^a^ p-value for RBC folate tertiles differences between cases and unrelated controls; ^b^ p-value for folate tertiles differences between cases and related controls; ^c^ p-value for RBC folate tertiles differences among cases, related and unrelated controls
